# Newborn screening for spinal muscular atrophy: The views of affected families and adults

**DOI:** 10.1002/ajmg.a.38220

**Published:** 2017-04-04

**Authors:** Felicity K. Boardman, Philip J. Young, Frances E. Griffiths

**Affiliations:** ^1^ Division of Health SciencesWarwick Medical SchoolUniversity of WarwickCoventryUnited Kingdom; ^2^ School of Life SciencesUniversity of WarwickCoventryUnited Kingdom

**Keywords:** bloodspot, ethics, newborn genetic screening, social implications, spinal muscular atrophy

## Abstract

Spinal muscular atrophy (SMA) is one of the leading genetic causes of infant death worldwide. However, due to a lack of treatments, SMA has historically fallen short of Wilson‐Jungner criteria. While studies have explored the acceptability of expanded newborn screening to the general public, the views of affected families have been largely overlooked. This is in spite of the potential for direct impacts on them and their unique positioning to consider the value of early diagnosis. We have previously reported data on attitudes toward pre‐conception and prenatal genetic screening for SMA among affected families (adults with SMA [*n* = 82] and family members [*n* = 255]). Here, using qualitative interview [*n* = 36] and survey data [*n* = 337], we report the views of this same cohort toward newborn screening. The majority (70%) of participants were in favor, however, all subgroups (except adults with type II) preferred pre‐conception and/or prenatal screening to newborn screening. Key reasons for newborn screening support were: (1) the potential for improved support; (2) the possibility of enrolling pre‐symptomatic children on clinical trials. Key reasons for non‐support were: (1) concerns about impact on the early experiences of the family; (2) inability to treat. Importantly, participants did not view the potential for inaccurate typing as a significant obstacle to the launch of a population‐wide screening program. This study underscores the need to include families affected by genetic diseases within consultations on screening. This is particularly important for conditions such as SMA which challenge traditional screening criteria, and for which new therapeutics are emerging.

## 1 INTRODUCTION

With recent developments in the field of genomics, for example, the increasing move toward next‐generation sequencing in various aspects of healthcare (Soden et al., 2014) and reproduction (Dondorp et al., 2015) newborn screening practices are facing new challenges both in the United Kingdom and beyond (Botkin, 2016; Botkin & Rothwell, 2016; Botkin et al., 2016). Originally introduced in the United Kingdom in the 1950s with the primary purpose of offering early treatment for babies with the metabolic disorder Phenylketonuria (where early intervention drastically alters outcomes), newborn screening has not significantly altered in the United Kingdom since this time, despite the introduction of new techniques and approaches (e.g. Guthrie's bloodspot technique/tandem mass spectrometry). Indeed, the list of conditions for which newborns are currently screened for within the United Kingdom (nine) remains modest compared to other European countries, or the United States, where in some states (e.g. Massachusetts), upwards of 60 conditions are screened for simultaneously (Downing & Pollitt, 2008). The inconsistent application of genetic screening in the international arena has been attributed to the lack of clear screening criteria. It is increasingly acknowledged that traditional Wilson‐Jungner criteria (now over 40 years old) do not adequately accommodate the very specific challenges posed by genetic disorders (Andermann, Blancquaert, Beauchamp, & Dery, 2008). In response to this, various attempts have been made to develop focused genetic screening criteria, however, uptake has been inconsistent and there appears to be no universally accepted standards to appraising potential genetic screening programs (Cornel et al., 2012; Walters, 1992).

As the criteria used to guide genetic screening policies come under scrutiny, the views and perspectives of stakeholder groups set to be affected by them have gained significance. Various studies have been undertaken exploring attitudes to expanded newborn screening, however, these have tended to focus on the views of clinicians (Hiraki, Ormond, Kim, & Ross, 2006) and/or (expectant) parents (e.g., Hasegawa, Fergus, Ojeda, & Au, 2011), with far less attention paid to the views of families living with the conditions that are potential screening candidates (with a few notable exceptions: Fragile X (Skinner, Sparkman, & Bailey, 2003), Mucopolysaccharidoses (Hayes, Collins, Sahhar, Wraith, & Delatycki, 2007), and Duchenne/Becker Muscular Dystrophy (Wood et al., 2014). This lack of consultation with affected families is surprising given that they are set to be directly impacted by the introduction of newborn screening, both through the change in public profile of the disease, but also through potential advances in research as affected children come to be enrolled earlier (and potentially pre‐symptomatically) onto clinical trials. Aside from these impacts, families living with potentially screened‐for conditions are also in a privileged position to consider the impact that an early diagnosis would have had for their lives, and consequently have much to offer studies considering the effects and desirability of expanded newborn screening (Wood et al., 2014).

This paper addresses this identified gap in literature by presenting attitudes toward newborn genetic screening (NGS) among families and individuals living with a condition for which NGS could feasibly soon be offered—Spinal Muscular Atrophy (SMA) (Phan, Taylor, Hannon, & Howell, 2015; Swoboda, 2010). Indeed, in light of emerging therapies for SMA, NGS for the condition is receiving renewed interest, evidenced by the formation of the “newborn screening working group” and the submission of SMA for consideration by the federal Recommended Uniform Screening Panel (RUSP) in early 2017. SMA is a neuromuscular disorder and one for which NGS has been described as particularly critical, not only because of the serious impact SMA has on families (Klug et al., 2016) and the acknowledged difficulties with obtaining a timely diagnosis (Lin, Kalb, & Yeh, 2015), but also because developing treatments for the condition requires children to be entered into clinical trials prior to the onset of symptoms, which is typically early in life (Prior & Nagan, 2016; Swoboda, 2010). While a limited number of studies have been conducted to explore public attitudes toward NGS for SMA (Rothwell, Anderson, Swoboda, Stark, & Botkin, 2013), there is very little evidence on the views of affected families, bar one study which included the views of five parents of SMA‐affected children (Wood et al., 2014).

Spinal Muscular Atrophy (SMA) is an autosomal recessive neuromuscular disorder and is a leading genetic cause of infant death (Munsat & Davies, 1992). Although presenting symptoms are due to the loss of the alpha motor neurones of the spinal cord (Munsat & Davies, 1992), recent reports have shown more systemic pathology (Somers et al., 2016; Thomson et al., 2016). It is sub‐classified into four main types, based on age of onset, severity, and inability to reach defined motor milestones (Munsat & Davies, 1992; Prior & Nagan, 2016; Prior & Russman, 1993; Prior, Nagan, Sugarman, Batish, & Braastad, 2011). Type I SMA is the severe form, with onset within the first few months of life and death usually occurring before 18 months through respiratory failure (Munsat & Davies, 1992). Type II SMA (intermediate) is the most divergent form, with onset usually within the first 2 years of life (Munsat & Davies, 1992). The impact on lifespan for individuals living with type II is dictated by the degree of respiratory involvement, with affected individuals facing end of life events anywhere from adolescence to late adulthood. Although mildly progressive, type II disease pathways tend to involve long “static” periods where symptoms do not change significantly (Glanzman et al., 2011; Munsat & Davies, 1992). Type III SMA is usually diagnosed after the age of 4 years, with the majority of able to sit and stand unaided (Dunaway et al., 2012; Glanzman et al., 2011; Munsat & Davies, 1992; Oh, Kim, Shim, & Sunwoo, 2011). Type IV SMA is diagnosed in adulthood, with patients developing generalized muscle weakness (Clermont et al., 1995). In both type III and IV there is a gradual deterioration in abilities over time, although life span is usually unaffected (Burglen et al., 1995; Clermont et al., 1995; Munsat & Davies, 1992).

We have previously reported data from the SMA Screening Survey (UK), which tested the views of 337 adults associated with SMA on three separate screening programs for the condition: (1) PCGS; (2) PNGS; and (3) NGS (Boardman, Young, & Griffiths, 2017). Our initial study reported the data on PCGS and PNGS; here we report the cohort's views on NGS. To the best of our knowledge, this is the largest study to date to systematically describe the views of SMA‐affected families and adults toward NGS. We also explore their perceptions of the key social and ethical concerns which currently surround NGS more broadly.

## 2 MATERIALS AND METHODS

An exploratory sequential mixed methods research design was adopted to address the complex and multi‐faceted question of screening for SMA. This design involved the use of qualitative interviews (*n* = 36) which were used to inform the development of a survey which was subsequently administered to a larger sample of families and adults with SMA (*n* = 337), as set out below.

### 2.1 Qualitative interviews

In‐depth qualitative interviews were conducted with 36 people who either have SMA or have SMA in their family between January and May 2014, with ethical approval for the study being granted by the Biomedical and Scientific Research Ethics Committee in early January 2014. Participants were recruited through advertisements placed in the newsletter of the main support and advocacy group for families living with SMA in the UK, SMA Support UK. The interviews were designed to explore experiences with SMA, views around and previous/anticipated use of reproductive genetic technologies, as well as perceptions of NGS for SMA.

Interviews were either completed over the telephone (*n* = 31) or face‐to‐face (*n* = 5), depending on participant preference and geographical location. The interview recordings were transcribed verbatim (with names and identifiers removed or changed) and the data analyzed using qualitative data analysis software, Nvivo10. A constructivist approach to grounded theory data analysis was used in order that the participants’ own meanings and interpretations guided the analysis, rather than those of the researcher. Initially, “open coding” of the data was carried out which was largely descriptive, before hierarchical coding was undertaken. A process of coding, refinement of concepts (through data interpretation), followed by re‐coding was carried out over a period of 5 months until “theoretical saturation” had occurred (Glaser, 1967). The qualitative analysis was completed by an experienced qualitative researcher, under the supervision of two senior academic mentors with expertise in qualitative methodology.

### 2.2 SMA Screening Survey (UK)

The SMA Screening Survey (UK) was developed directly from the qualitative data in order to ensure that the priorities of SMA families were reflected in the survey questions. The survey assessed views on PCGS, PNGS, and NGS. The survey was developed through single sentence “attitude/belief” statements derived from the qualitative interviews, which were in turn developed into quantitative survey questions through the use of a Likert scale. As such, the seven key themes from the qualitative analysis were directly used to delineate the key domains of the survey. In this way, the qualitative analysis directly informed the content of the survey (see Table 1 for a list of statements). Questions designed to capture demographic information from respondents (such as educational attainment, religious faith, and ethnicity) were either directly replicated from, or appear as modified versions of, questions used in the 2011 UK Census survey.

**Table 1:** Response summaries for questions assessing views on newborn genetic screening

		Family subgroups					Patient subgroups			
Question	All responders (*n *= 337)	Families (*n *= 255)	Type families (*n *= 120)	Type II/III families (*n *= 109)	Type II families (*n *= 87)	Type III families (*n *= 22)	Patients (*n *= 82)	Type II and III patients (*n *= 58)	Type II patients (*n *= 27)	Type III patients (*n *= 31)
Identifying SMA at birth would lead to better support for children and families										
Agree	282 (84%)	206 (81%)	101 (84%)	84 (77%)	66 (76%)	18 (82%)	76 (93%)	54 (93%)	27 (100%)	27 (87%)
Other	55 (16%)	49 (19%)	19 (16%)	25 (23%)	21 (24%)	4 (18%)	6 (7%)	4 (7%)	0 (0%)	4 (13%)
Identifying SMA at birth would extend life expectancy of SMA children										
Agree	127 (38%)	86 (34%)	40 (33%)	41 (38%)	32 (37%)	9 (41%)	41 (50%)	34 (59%)	20 (74%)	14 (45%)
Other	210 (62%)	169 (66%)	80 (67%)	68 (62%)	55 (63%)	13 (59%)	41 (50%)	24 (41%)	7 (26%)	17 (55%)
Identifying SMA at birth and not during pregancy removes parents ability to make informed decisions about bringing SMA children into the world										
Agree	192 (57%)	153 (60%)	74 (62%)	62 (57%)	48 (55%)	14 (64%)	39 (48%)	27 (47%)	11 (41%)	16 (52%)
Other	145 (43%)	102 (40%)	46 (38%)	47 (43%)	39 (45%)	8 (36%)	43 (52%)	31 (53%)	16 (59%)	15 (48%)
Identifying SMA before symptoms emerge will prevent families and chidren enjoying life while they are symptom free										
Agree	149 (44%)	123 (48%)	64 (53%)	49 (45%)	28 (32%)	11 (50%)	26 (32%)	16 (28%)	7 (26%)	9 (29%)
Other	188 (56%)	132 (52%)	56 (47%)	60 (55%)	49 (68%)	11 (50%)	56 (68%)	42 (72%)	20 (74%)	22 (71%)
Idenitfying SMA at birth wil help research by enabling more children to be enrolled into clinical trials early on										
Agree	251 (74%)	188 (74%)	88 (73%)	84 (77%)	68 (78%)	16 (73%)	63 (77%)	42 (72%)	21 (78%)	21 (68%)
Other	86 (26%)	67 (26%)	32 (23%)	25 (23%)	19 (22%)	6 (27%)	19 (23%)	16 (23%)	6 (22%)	10 (32%)
Identification of SMA at birth would interfere with the early bonding process										
Agree	50 (15%)	38 (15%)	17 (14%)	18 (17%)	18 (21%)	0 (0%)	12 (15%)	9 (16%)	5 (19%)	4 (13%)
Other	287 (85%)	217 (85%)	103 (86%)	91 (83%)	69 (79%)	22 (100%)	70 (85%)	49 (84%)	22 (81%)	27 (87%)
Identification of SMA at birth would make the diagnosis easier for parents accept										
Agree	100 (30%)	64 (26%)	30 (25%)	28 (26%)	22 (25%)	6 (27%)	36 (44%)	25 (43%)	10 (37%)	15 (48%)
Other	237 (70%)	191 (74%)	90 (75%)	81 (74%)	65 (75%)	16 (73%)	46 (56%)	33 (57%)	17 (63%)	16 (52%)
Identifying SMA at birth would spare the difficulties associated with finding a diagnosis for a child later on										
Agree	222 (66%)	161 (63%)	77 (64%)	68 (62%)	50 (57%)	18 (82%)	61 (74%)	44 (76%)	22 (81%)	22 (71%)
Other	115 (34%)	94 (37%)	43 (36%)	41 (38%)	37 (43%)	4 (18%)	21 (26%)	14 (24%)	5 (19%)	9 (29%)
Identifying SMA at birth is important, even if the type can not be determined										
Agree	225 (67%)	161 (63%)	79 (66%)	64 (59%)	49 (56%)	15 (68%)	64 (79%)	45 (78%)	20 (74%)	25 (81%)
Other	112 (33%)	94 (37%)	41 (34%)	45 (41%)	38 (44%)	7 (32%)	18 (21%)	13 (22%)	7 (26%)	6 (19%)
Identifying SMA at birth is important because it wil enable parents to make informed decisions about future pregnancies										
Agree	272 (81%)	205 (80%)	101(84%)	85 (78%)	68 (78%)	17 (77%)	67 (82%)	48 (83%)	22 (81%)	26 (84%)
Other	65 (19%)	50 (20%)	19 (16%)	24 (22%)	19 (22%)	5 (23%)	15 (18%)	10 (17%)	5 (19%)	5 (16%)
It is un ethical to screen newborns for conditions that have no effective treatmen										
Agree	27 (8%)	19 (7%)	8(6%)	11 (10%)	9 (10%)	2 (9%)	8 (10%)	5 (8%)	3 (11%)	2 (6%)
Other	310 (92%)	236 (93%)	112 (94%)	98 (90%)	78 (90%)	20 (91%)	74 (90%)	53 (92%)	24 (89%)	29 (94%)
I woulb support a Newborn screening program for SMA										
Agree	236 (70%)	175 (68.6%)	84 (70%)	72 (66%)	57 (66%)	15 (68%)	61 (74.4%)	45 (78%)	21 (78%)	24 (77%)
Other	101 (30%)	80 (31.4%)	36 (30%)	37 (34%)	30 (37%)	7 (32%)	21 (25.6%)	13 (22%)	6 (22%)	7 (23%)

Response breakdowns are shown for family subgroups (all, type I, type II/III combined, type II and type III) and patient subgroups (all, type II/III combined, type II and type III). Responses for each question were stratified as “agree” versus “other” (other = disagree and neither disagree nor agree). This stratification was used to enable binominal logistic regression of each subgroup.

As well as the underpinning qualitative work, the survey was also passed through three expert panels, made up of professionals working with families affected by SMA (SMA Support UK/SMA Patient Registry) as well as people living with SMA themselves. Ethical approval for the survey was granted (separately to that for the qualitative interviews) by the Biomedical and Scientific Research Ethics Committee in July 2014.

Quantitative data collection was carried out over a period of 10 months, from September 1st, 2014 to June 30th, 2015. Two versions of the survey were made available, an online version (hosted on a secure website) and a paper copy. The survey was made available online via UK SMA Support and the Imaging Future research website.

Potential participants were invited to complete the survey if they were aged 18 or over and either had SMA themselves, or at least one diagnosis of SMA in the family. People affected by one the variant forms of SMA (Spinal Muscular Atrophy and Respiratory Distress, Spinal Bulbar Muscular Atrophy) were also invited to take part. No restrictions were placed on the type of family members invited to take part: step‐, adopted and fostered family members were included. The recruitment strategy for family members was kept broad (and included non‐biological relatives) as the social relationship to the person with SMA was considered as important as the biological relatedness of the person. While the SMA Screening Survey (UK) also included questions on PCGS and PNGS, due to the very specific social and ethical issues pertaining to these types of screening (i.e., those of selective reproduction), data on attitudes to NGS are the focus of this paper. Data on the other screening programs are discussed elsewhere (Boardman et al., 2017).

### 2.3 Statistical analysis

The attitudes of families and adults with SMA toward NGS were compared to determine if there were any statistical differences. The following subgroup analyses were performed: All participants were analyzed collectively to identify any overriding trends (all participants). Responses from families (all) and adults with SMA (all) were compared to determine if living with the disease directly altered views. Sub‐analyses on participants associated with the three most prevalent childhood forms of SMA (types I, II, and III) were then performed. Responses from families associated with type I were compared with responses from families with milder forms (type II/III SMA (combined), type II alone, and type III alone)‐ to determine if severity altered families’ views. Responses were compared between families and adults with SMA, to determine if the relationship to SMA affects views (when severity is standardized). This analysis was split into three: (1) type II‐associated participants; (2) type III‐associated participants; and (3) type II/III combined (the combined analysis was performed to facilitate logistic regression analysis based on the relatively low number of adults with SMA in the two subgroups. Finally, responses from adults with type II were compared to adults with type III, and responses form type II families were compared to type III families. This assessed whether the severity and age of diagnosis impacts views, and whether any differences were seen in both families and adults living with the disease. For the subgroup analysis, families members associated with more than one form of the disease were classified according the most severe form within their family (e.g., a family with a type I and type II child would be classified as a type I family).

In each of the subgroup analyses, the individual questions were assessed and then responses correlated against support for screening. For each question the number of “agree” vs. “other” responses were reported and statistical differences between the subgroups were assessed using a chi‐squared analysis (Graphpad Prism software, v6). Associations between positive “agree” responses to each question were assessed using binary logistic regression (performed against survey Q20l (I would support a newborn genetic screen for SMA). Logistic regression was performed using SPSS v22 (IBM).

## 3 RESULTS

The cohort characteristics have been previously reported (Boardman et al., 2017). Briefly, of the 337 participants, 255 were family members of people with SMA (75.7%) and 82 had SMA themselves (24.3%). Most participants were female (74.4%); aged between 35 and 55 years (52%); were not educated to degree level (63.8%); were religious (55%); were parents (82%); had lived/were living with someone with SMA (82%) and had experience with SMA types 0, I, or II (69.4%). The remainder of the sample (31.6%) were affected by rarer forms of SMA (e.g., type IV).

Overall, 70% of survey participants were in favor of NGS, with no statistical differences between any of the analyzed sub‐groups (Tables 1 and 2). However, the overall levels of support were lower than the previously reported levels of support in the same participants for both PCGS (77%) and PNGS (76%) (Boardman et al., 2017).

**Table 2:** Statistical analysis response summaries for questions assessing views on newborn genetic screening

	Statistical comparison (chi‐squared analysis)								
	Families (all) vs. patients (all)	Type 1 vs. 11/III (families)	Type 1 vs. II (families)	Type 1 vs. III (families)	Type II vs. III (families)	Type II vs. III (patients)	Type 11/III (Families vs. patients)	Type II (Families vs. patients)	Type III (Families vs. patients)
Question	*p* value	*p* value	*p* value	*p* value	*p* value	*p* value	*p* value	*p* value	*p* value
Identifying SMA at birth would lead to better support for children and families	**0.01**	0.18	0.15	0.75	0.77	0.11	**0.009**	**0.003**	0.71
Agree									
Other									
Identifying SMA at birth would extend life expectancy of SMA children	**0.008**	0.58	0.65	0.62	0.81	**0.03**	**0.01**	**0.0009**	0.79
Agree									
Other									
Identifying SMA at birth and not during pregancy removes parents ability to make informed decisions about bringing SMA children into the world	**0.04**	0.51	0.39	0.86	0.63	0.44	0.25	0.27	0.41
Agree									
Other									
Identifying SMA before symptoms emerge will prevent families and children enjoying life while they are symptom free	**0.009**	0.23	**0.02**	0.81	0.32	0.79	**0.03**	0.35	0.15
Agree									
Other									
Idenitfying SMA at birth will help research by enabling more children to be enrolled into clinical trials early on	0.57	0.54	0.51	0.95	0.57	0.55	0.57	0.96	0.76
Agree									
Other									
Identification of SMA at birth would interfere with the early bonding process	0.95	0.71	0.26	0.07	**0.02**	0.72	0.86	0.81	0.07
Agree									
Other									
Identification of SMA at birth would make the diagnosis easier for parents to accept	**0.001**	0.91	0.96	0.79	0.84	0.43	**0.02**	0.32	0.15
Agree									
Other									
Identifying SMA at birth would spare the difficulties associated with finding a diagnosis for a child later on	0.06	0.78	0.38	0.14	**0.04**	0.37	0.08	**0.03**	0.51
Agree									
Other									
Identifying SMA at birth is important, even if the type can not be determined	**0.01**	0.27	0.19	0.83	0.34	0.75	**0.01**	0.11	0.34
Agree									
Other									
Identifying SMA at birth is important because it will enable parents to make informed decisions about future pregnancies	0.79	0.24	0.28	0.53	0.92	0.81	0.54	0.79	0.72
Agree									
Other									
It is unethical to screen newborns for conditions that have no effective treatment	0.51	0.47	0.44	0.65	0.86	0.52	0.75	0.91	0.72
Agree									
Other									
I would support a newborn screening program for SMA	0.61	0.57	0.54	0.86	0.81	0.97	0.15	0.34	0.53
Agree									
Other									

Response breakdowns are shown for family subgroups (all, type I, type II/III combined, type II, and type III) and patient subgroups (all, type II/III combined, type II and type III). Responses for each question were stratified as “agree” versus “other” (other = disagree and neither disagree nor agree). Response distributions were compared using chi‐squared analysis (*p*‐value; significant differences are highlighted in bold (*p *< 0.05).

Interestingly, while the majority of participants agreed that NGS was important because it would lead to better support for children and families, would extend life expectancy, would help research by enabling children to enrol on clinical trials earlier and would prevent the difficulties for a child associated with a later diagnosis (Tables 1 and 2), there were differences between the individual subgroups. Fewer family members than adults with SMA believed NGS would result in better support (81% vs. 93%, *p* = 0.01; Tables 1 and 2); this difference was predominantly due to differences seen between type II families and adults living with type II SMA (76% vs. 100%, *p* = 0.009; Tables 1 and 2). There was also a considerable dichotomy between families and adults with SMA regarding the expectation of extended life expectancy, with fewer type II family members thinking it would increase life years compared to adults diagnosed with type II (37% vs. 74%; *p* = 0.01; Tables 1 and 2). Notably, there were also fewer type III patients than type II patients who thought life expectancy would increase (45% vs. 74%, *p* = 0.0009; Tables 1 and 2). In comparison, there was general uniform agreement across all subgroups that NGS would enable early enrollment on clinical trials and that it would enable parents to make informed decisions about future pregnancies (Tables 1 and 2).

A lower proportion of type II family members thought NGS would spare them some of the difficulties associated with a later diagnosis for the child (57%); this was significantly lower than for adults with type II SMA (81%; *p* = 0.03; Tables 1 and 2) and type III families (82%; *p* = 0.04; Tables 1 and 2). In addition, proportionately more families associated type I SMA compared with type II families thought that an earlier diagnosis would prevent families enjoying life before symptoms emerge (53% vs. 32%; *p* = 0.02; Tables 1 and 2).

One of the key questions surrounding NGS for SMA is whether NGS can still offer useful information, even without the ability to accurately diagnose SMA type. This is one of the central reasons why the UK National Screening Committee (NSC) rejected instigation of an SMA screening program in the United Kingdom. With this in mind, it is important to note that the majority of participants from all subgroups thought the importance of an early diagnosis out‐weighed the accurate ability to type (using current methods). However, support was generally lower in families versus adults with SMA (63% vs. 79%, *p* = 0.01; Tables 1 and 2); although the differences were not significant when type II and III individual comparisons were made. Indeed, when these groups were merged there were significantly fewer type II/III families than adults with type II/III who thought diagnosis at birth was important when accuracy regarding type could not be guaranteed (59% vs. 78%; *p* = 0.01; Tables 1 and 2).

Univariate logistic regression analysis confirmed the direct comparison analysis (Tables 3 and 4). All family subgroups who supported NGS generally thought it would improve support, extend life expectancy, enable early enrollment on clinical trials, would make the diagnosis easier for parents to accept, spare difficulties associated with a later diagnosis, and allow informed decisions regarding future pregnancies (indicated by a positive odds ratio; *p* < 0.05; Table 3). In comparison, while adults with type II/III (combined subgroup) agreed it would lead to better support and allow informed decisions for future pregnancies, there was no general agreement that it would increase life expectancy, allow early enrollment on trials (although this was approaching significance; *p* = 0.09; Table 4), would make diagnosis easier to accept or would spare children the difficulties associated with a later diagnosis (Table 4). All adult and family subgroups in favor of NGS predominantly thought it was important, even if type could not be determined (Tables 3 and 4). Regarding negative drivers, participants in favor of NGS did not agree that it was unethical (as there is no therapy) or that it would interfere with the early bonding process; this was consistent for all subgroups analyzed where there were enough responses to perform a statistically relevant logistic regression (Tables 3 and 4).

**Table 3:** Logistic regression analysis highlighting positive and negative drivers associated with responders views on newborn genetic screening

	Family subgroups											
	All		F (all)		Type 1 F; *n* = 120)		Type II and III F; *n* = 109)		Type II F; *n* = 87)		Type III F; *n* = 22)	
Question	Odds ratio (95% Cl)	*p*‐value	Odds ratio (95%Cl)	*p*‐value	OR (95%Cl)	*p*‐value	OR (95%Cl)	*p*‐value	OR (95%Cl)	*p*‐value	OR (95%Cl)	*p*‐value
Identifying SMA at birth would lead to better support for children and famlies		**<0.0001**		**<0.0001**		**<0.0001**		**<0.0001**		**<0.0001**		0.06
Other	**Reference**		**Reference**		**Reference**		**Reference**		**Reference**		Reference	
Agree	**18.16 (8.62–38.25)**		**2194 (9.53–50.52)**		**36.68 (7.80–162.45)**		**15.76 (5.16–48.08)**		**17.32 (4.98–60.27)**		10.50 (0.84–130.66)	
Identifying SMA at birth would extend life expectancy of SMA children		**<0.0001**		**<0.0001**		**0.005**		**0.005**		**0.02**		0.11
Other	**Reference**		**Reference**		**Reference**		**Reference**		**Reference**		Reference	
Agree	**3.10 (1.80–5.35)**		**4.15 (2.09–8.22)**		**4.42 (1.56–12.52)**		**3.83 (1.49–9.85)**		**3.35 (119–9.44)**		6.85 (0.65–71.72)	
Identifying SMA at birth and not during pregancy removes parents ability to make informed decisions about bringing SMA children into the world		**0.02**		**0.02**		0.62		**0.04**		0.11		0.17
Other	**Reference**		**Reference**		Reference		**Reference**		Reference		Reference	
Agree	**1.72 (1.08–276)**		**1.82 (1.06–3.11)**		1.22 (0.55–2.71)		**2.32 (1.03–5.21)**		0.08 (0.84–5.10)		3.66 (0.55–24.13)	
Identifying SMA before symptoms emerge will prevent families and children enjoying life while they are symptom free		0.87		0.87		0.38		0.57		0.61		**0.04**
Other	Reference		Reference		Reference		Reference		Reference		**Reference**	
Agree	1.03 (0.64–166)		1.04 (0.61–1.77)		1.42 (0.64–3.10)		0.78 (0.36–1.76)		1.25 (0.51–3.08)		**0.08 (0.008–0.89)**	
Identifying SMA at birth will help research by enabling more children to be enrolled into clinical trials ealy on		**<0.0001**		**<0.0001**		**<0.0001**		**<0.0001**		**<0.0001**		**0.008**
Other	**Reference**		**Reference**		**Reference**		**Reference**		**Reference**		**Reference**	
Agree	**6.30 (3.69–10.74)**		**8.07 (4.33–15.03)**		**6.10 (2.52–14.74)**		**11.61 (4.04–33.36)**		**9.10 (2.83–29.16)**		**35.00 (2.57–475.31)**	
Identification of SMA a birth would interfere with the early bonding process		**<0.0001**		**<0.0001**		**0.002**		**0.03**		**0.03**		0.99
Other	**Reference**		**Reference**		**Reference**		**Reference**		**Reference**		Reference	
Agree	**0.26 (0.14–0.49)**		**0.27 (0.13–0.54)**		**0.17 (0.05–0.52)**		**0.33 (0.12–0.94)**		**0.32 (0.11–0.94)**		NR*	
Identification of SMA a birth would make the diagnosis easier for parents to accept		**<0.0001**		**<0.0001**		**0.005**		**0.003**		**0.007**		0.99
Other	**Reference**		**Reference**		**Reference**		**Reference**		**Reference**		Reference	
Agree	**4.95 (251–9.76)**		**13.73 (4.15–45.35)**		**8.50 (1.90–37.96)**		**21.60 (2.79–166.69)**		**16.91 (2.14–133.37)**		NR*	
Identifying SMA at birth would spare difficulties associated with finding a diagnosis for child later on		**<0.0001**		**<0.0001**		**<0.0001**		**<0.0001**		**<0.0001**		0.99
Other	**Reference**		**Reference**		**Reference**		**Reference**		**Reference**		Reference	
Agree	**6.71 (4.02–1122)**		**9.23 (5.04–16.92)**		**4.71 (2.05–10.83)**		**14.12 (5.39–36.93)**		**13.53 (456–40.17)**		NR*	
Identifying SMA at birth is important, even if the type can not determined		**<0.0001**		**<0.0001**		**<0.0001**		**<0.0001**		**<0.0001**		**0.003**
Other	**Reference**		**Reference**		**Reference**		**Reference**		**Reference**		**Reference**	
Agree	**22.09 (12.13–40.20)**		**28.25 (13.77–58.14)**		**15.00 (5.81–38.70)**		**62.84 (16.39–240.92)**		**65.80 (13.43–322.23)**		**84.00 (4.47–1576.51)**	
Identifying SMA at birth is important because it will enable parents to make informed decisions about future pregnancies		**<0.0001**		**<0.0001**		**<0.0001**		**<0.0001**		**<0.0001**		**0.02**
Other	**Reference**		**Reference**		**Reference**		**Reference**		**Reference**		**Reference**	
Agree	**11.66 (6.21‐21.89)**		**14.18 (6.68–30.07)**		**10.55 (3.26–30.97)**		**14.14 (4.64–43.09)**		**13.25 (3.82–45.92)**		**18.66 (1.50–232.29)**	
It is unethical to screen newborns for conditions that have no effective treatment		**<0.0001**		**0.001**		**0.01**		**0.03**		**<0.0001**		**0.02**
Other	**Reference**		**Reference**		**Reference**		**Reference**		**Reference**		**Reference**	
Agree	**0.12 (0.05‐0.30)**		**0.18 (0.06–0.50)**		**0.122 (0.02–0.63)**		**025 (0.06–0.92)**		**13.25 (3.82–45.92)**		**18.66 (1.50–232.29)**	

Odds ratios are presented for response breakdowns are shown for family sub‐groups (all, type I, type II/III combined, type II and type III). Responses for each question were stratified as “agree” versus “other” (other = disagree and neither disagree nor agree). Odds ratios (OR) show the likelihood that responders who agreed with each individual question (variable) were also in favor of newborn genetic screening. Positive drivers are indicated by a odds ratio >1 and a *p‐*value <0.05); negative drivers are indicated by an odds ration <1 and a *p‐*value <0.05). Significant drivers are highlighted in bold. Several ORs are presented as NR (not returned); this is because of the low number of responders and included “other” responses.

**Table 4:** Logistic regression analysis highlighting positive and negative drivers associated with responders views on newborn genetic screening

	Adult with SMA (AwS) sub‐groups							
	AwS (all)		Type II and III AwS; *n *= 58)		Type II AwS; *n *= 27)		Type III AwS; *n *= 31)	
Question	Odds ratio (95%Cl)	*p*‐value	OR (95%Cl)	*p*‐value	OR (95%Cl)	*p*‐value	OR (95%Cl)	*p*‐value
Identifying SMA at birth would lead to better support for children and families		**0.03**		**0.03**		0.99		**0.02**
Other	**Reference**		**Reference**		Reference		**Reference**	
Agree	**6.94 (1.16–41.20)**		**13.20 (1.24–140.51)**		NR*		**17.25 (1.41–210.12)**	
Identifying SMA at birth would extend life expectancy of SMA children		0.44		0.31		0.64		0.32
Other	Reference		Reference		Reference		Reference	
Agree	1.47 (0.54–3.99)		1.92 (0.55–6.67)		1.60 (0.22–11.49)		2.50 (0.40–15.50)	
Identifying SMA at birth and not during pregnancy removes parents ability to make informed decisions about bringing SMA children into the world		0.31		0.06		0.67		**0.04**
Other	Reference		Reference		Reference		**Reference**	
Agree	1.67 (0.61–4.63)		3.81 (0.92–15.71)		1.50 (0.22–10.07)		**10.00 (1.03–97.04)**	
Identifying SMA before symptoms emerge will prevent families and children enjoying life while they are symptom free		0.72		0.11		0.56		0.99
Other	Reference		Reference		Reference		Reference	
Agree	1.22 (0.41–3.16)		6.00 (0.71–50.59)		2.00 (0.19–20.89)		NR*	
Identifying SMA at birth will help research by enabling more children to be enrolled into clinical trials early on		0.C6		0.09		0.08		0.49
Other	Reference		Reference		Reference		Reference	
Agree	2.79 (0.935–8.37)		3.00 (0.82–10.97)		6.00 (0.80–44.94)		1.82 (0.32–10.34)	
Identification of SMA at birth would interfere with the early bonding process		**0.045**		0.09		0.31		0.18
Other	**Reference**		Reference		Reference		Reference	
Agree	**0.27 (0.77–0.96)**		0.28 (0.06–1.26)		0.33 (0.04–2.69)		0.22 (0.02–2.02)	
Identification of SMA at birth would make the diagnosis easier for parents to accept		0.53		0.31		0.46		0.06
Other	Reference		Reference		Reference		Reference	
Agree	1.37 (0.50–3.80)		1.96 (0.52–7.33)		0.50 (0.07–3.14)		8.40 (0.87–81.08)	
Identifying SMA at birth would spare the difficulties associated with finding a diagnosis for a child later on		0.13		0.17		0.99		**0.01**
Other	Reference		Reference		Reference		**Reference**	
Agree	2.27 (0.77–6.64)		2.50 (0.65–9.49)		NR*		**12.50 (1.76–88.73)**	
Identifying SMA at birth is important, even if the Type can not be determined		**<0.0001**		**<0.0001**		**0.02**		0.99
Other	**Reference**		**Reference**		**Reference**		Reference	
Agree	**12.22 (3.65–40.86)**		**46.66 (8.17–266.56)**		**12.00 (1.48–97.17)**		NR*	
Identifying SMA at birth is important because it will enable parents to make informed decisions about future pregnancies		**0.002**		**0.001**		**0.04**		**0.007**
Other	**Reference**		**Reference**		**Reference**		**Reference**	
Agree	**6.87 (2.05–22.98)**		**16.33 (3.29–80.92)**		**9.50 (1.09–82.72)**		**30.66 (2.51–373.54)**	
It is unethical to screen newborns for conditions that have no effective treatment		**0.002**		**0.01**		0.99		0.36
Other	**Reference**		**Reference**		Reference		Reference	
Agree	**0.03 (0.004–0.29)**		**0.05 (0.005–0.51)**		NR*		0.26 (0.01–4.80)	

Odds ratios are presented for response breakdowns are shown for adults with SMA sub‐groups (type II/III combined, type II and type III). Responses for each question were stratified as “agree” versus “other” (other = disagree and neither disagree nor agree). Odds ratios (OR) show the likelihood that responders who agreed with each individual question (variable) were also in favor of newborn genetic screening. Positive drivers are indicated by a odds ratio >1 and a *p‐*value <0.05); negative drivers are indicated by an odds ration <1 and a *p‐*value <0.05). Significant drivers are highlighted in bold. Several ORs are presented as NR (not returned); this is because of the low number of responders and included “other” responses.

We compared the levels of support for NGS against support for two alternative programs (PCGS and PNGS). As reported here and elsewhere (Boardman et al., 2017), in general there is more support for PCGS and PNGS than NGS in all analyzed subgroups (Table 5). The kappa analysis suggests there is a minimal‐weak agreement within each subgroup; this is important, because it highlights that participants are not simply infavor of all tests, instead there are subtle differences between the different groups that reflect their views and experiences. As highlighted in the analysis, adults with type II SMA are the only subgroup that preferentially support NGS over the other groups (Table 5). This is in keeping with our previous report, which demonstrates these participants have a comparatively positive view on their condition, believing they have fulfilling lives and can have a valuable impact on society (Boardman et al., 2017). Therefore, their support for NGS is understandable, because it is the one test that would not result in fewer children with type II SMA being born (this was highlighted in our previous study as one of the main reasons adults with type II SMA were opposed to PCGS and PNGS programs) (Boardman et al., 2017).

**Table 5:** Levels of comparative support for the three potential SMA screening programs (newborn screening, pre‐conception genetic screening, and prenatal screening)

	I woud support a newborn screening program			
Question	Other	Agree	Kappa	*p*‐value
Type I families (*n* = 120)				
I would support a pre‐conception genetic screening program			0.28	<0.0001
Other	10 (8%)	4 (3%)		
Agree	26 (22%)	80 (67%)		
I would support a prenatal screening program			0.31	<0.0001
Other	11 (9%)	4 (3%)		
Agree	25 (21%)	80 (67%)		
Type II families (*n* = 87)				
I would support a pre‐conception genetic screening program			0.25	0.01
Other	13 (15%)	11 (13%)		
Agree	17(20%)	46 (53%)		
I would support a prenatal screening program			0.39	<0.0001
Other	16 (18%)	9 (10%)		
Agree	14 (16%)	48 (56%)		
Type III families (*n* = 22)				
I would support a pre‐conception genetic screening program			0.23	0.26
Other	3 (14%)	3 (14%)		
Agree	4 (18%)	12 (54%)		
I would support a prenatal screening program			0.58	0.006
Other	5 (23%)	2 (9%)		
Agree	2 (9%)	13 (59%)		
Adults with type II SMA (*n* = 27)				
I would support a pre‐conception genetic screening program			0.48	0.01
Other	5 (19%)	5 (19%)		
Agree	1 (4%)	16 (58%)		
I would support a prenatal screening program			0.32	0.05
Other	5 (19%)	8 (30%)		
Agree	1 (4%)	13 (47%)		
Adults with type III SMA (*n* = 31)				
I would support a pre‐conception genetic screening program			0.24	0.16
Other	2 (6%)	2 (6%)		
Agree	5 (16%)	22 (72%)		
I would support a prenatal screening program			0.51	0.004
Other	4 (13%)	2 (6%)		
Agree	3 (9%)	22 (72%)		
Interpretation of cohen's kappa				
Kappa range		Interpretation		
0–0.2		No agreement		
0.21–0.39		Minimal agreement		
0.40–0.59		Weak agreement		
0.600.79		Moderate agreement		
0.800.90		Strong agreement		
>0.90		Almost perfect agreement		

Support was compared in the following sub‐groups: (1) type I families; (2) type II families; (3) type III families; (4) Adults with type II SMA; and (5) adults with type III SMA. Agreement was assessed using a kappa analysis‐cohen's interpretation criteria are included; statistical significance of the kappa (*p*‐value) is shown (significance assigned using a <0.05 cut off).

## 4 DISCUSSION

Screening newborns for conditions in the absence of effective treatments has been described as ethically problematic, not least because the direct benefits to the child of undergoing such screening are limited (Schmidt et al., 2012;Timmermans & Buchbinder, 2010; Tluczek, Orland, & Cavanagh, 2011). Moreover, NGS carries multiple risks for that child, not only in terms of the widely discussed (and sometimes long‐term) physical and psychological risks of indeterminate or false positive/negative results (Schmidt et al., 2012; Timmermans & Buchbinder, 2010; Tluczek et al., 2011), but also in terms of the inherent risks of clinical trial enrollment in relation to experimental therapies. It is noteworthy, therefore, that for the majority of people who participated in this study, the possibility of facilitating clinical trials was seen as a positive reason to support screening. This support of trials was fairly even across all types of SMA as well as between family members and adults with SMA (Table 1). It is unclear whether participants perceived a direct benefit to trial enrollment for SMA children or whether they accepted the indirect benefits. However, the importance of supporting such trials, as well as the earlier introduction of support and healthcare, the importance of an earlier diagnosis, and the benefits in terms of future reproductive decisions all featured as positive drivers for NGS support (Table 1).

The importance of an early SMA diagnosis and trial enrollment has received increased attention recently following the preliminary reports from a phase 2, open‐label, dose‐escalation study of Nusinersen (an antisense oligonucleotide that modifies *SMN2* RNA splicing) (Chiriboga et al., 2016; Finkel et al., 2016; Hache et al., 2016). The trial involved 20 participants, with 2–3 copies of *SMN2* and age of onset ranging from 21 to 154 days (Finkel et al., 2016). Data from this trial demonstrated that pre‐symptomatic infants at high genetic risk of type I SMA responded well to Nusinersen, achieving motor milestones in timelines more consistent with normal development (Finkel et al., 2016). These findings suggest that improved outcomes (motor function, achieved motor milestones, and increased time to ventilation) could be achieved if pre‐symptomatic patients (identified through NGS) could be enrolled and treated with Nusinersen (or similar ASOs). This therapeutic has been approved by the U.S. FDA and may be prescribed for newborns with high genetic risk for type I SMA.

For the 30% of the sample who were not openly in favor of NGS for SMA, concerns about parent‐child bonding and the ethics of a newborn program in the absence of treatments emerged as key reasons for their non‐support. The newborn screening literature highlights the detrimental impact that an unsought and serious diagnosis can have on the early parent‐child relationship in terms of bonding and levels of parental stress (al‐Jader, Goodchild, Ryley, & Harper, 1990; Grob, 2008). Given the gravity of an SMA diagnosis, the lack of available treatments and difficulties associated with accurate prognostic information, this was also an issue that emerged as significant for SMA families.

Concerns about the impact of NGS on the early experiences of the family were also evident in attitudes toward the impact of a pre‐symptomatic diagnosis. Significantly more type I and type III family members than any other subgroup agreed that NGS would prevent families from enjoying care‐free time with their baby before their SMA symptoms emerged. It is perhaps unsurprising that this issue was particularly pronounced for families living with type I and III, given the extremely curtailed life expectancy of infants with type I SMA and the relatively long period of time before the onset of symptoms in the case of type III SMA.

Subanalyses of families and adults with SMA reveal evidence to suggest that the reasons underpinning non‐support differed across the types, as well as between family members and adults with SMA. Family members with experience of type I SMA who did not want NGS did so not out of a rejection of screening per se, but rather because they wanted screening in a different form. In contrast, the data highlight that for 22% of adults with type II SMA rejected all forms of screening for SMA. It is noteworthy, however, that this view was not evidenced among adults with type III SMA, and seems to be related to the perceptions of the condition among adults with type II. Shakespeare postulates that people with fixed impairments from birth or early childhood are often better adjusted to their disabilities than those whose impairments are later onset, fluctuate, or involve periods of decline or deterioration (Shakespeare, 2006). For those who have always lived with their impairment, and set their lives up around its existence, the concept of screening and cure may be deemed secondary to the broader social and political goals of equality and an open, inclusive society for people with disabilities. It has recently been reported that adults with more clinically severe forms of SMA report higher quality of life and perceptions of the condition than those with milder and adult onset forms of SMA (Kruitwagen‐Van Reenen et al., 2016). Our study demonstrates that these differing perceptions of the condition emerged within our sample, but also that they translated into negative attitudes toward screening and SMA prevention.

In spite of this identified resistance among a subset of adults with type II SMA, NGS emerged from the SMA Screening Survey (UK) analysis as the least divisive of all the forms of screening explored. Indeed, the vast majority of participants were positive about NGS's potential to improve the lives of people with SMA. The fact that NGS elicited far less resistance among adults with type II SMA than did the other screening programs is likely because NGS is not primarily designed to reduce the number of births of children with SMA (Boardman et al., 2017). Rather, NGS lends itself to a model of disease prevention that relies on early identification and amelioration of disease symptoms rather than the more ethically complex approach of avoiding the births of affected individuals.

There are potential limitations in this study. Due to confidentiality and data protection issues, no identifiable data were asked of individuals who participated in the SMA Screening Survey (UK), including IP addresses (where the survey was completed online). This meant that there was no mechanism in place to prevent an individual completing multiple surveys. Moreover, there was no way of verifying that the participant fitted the inclusion criteria to participate in the survey. Participants were furthermore accessed through a national support group, personal networks, and a patient registry rather than neuromuscular clinics, which may have introduced bias. Due to the very poor prognoses associated with types 0 and I SMA, the adults with SMA who participated in the survey were largely affected with clinically milder forms of the disease (although two participating adults reported that they had a diagnosis of type I SMA, and all types of SMA can be associated with significant disability and disease burden). This may have impacted on how the disease was presented and the differences in perceptions of quality of life associated with SMA between adults living with it and parents of babies who died of types 0 or I SMA. Our analysis grouped participants as “families” or “adults with SMA.” This means we have not reported whether there are differences between close (parents, siblings) and distant (cousins, uncles etc) family members. This was because the low numbers involved for some of the family members reduced the significance of the analysis.

In conclusion, this study highlights that for families living with SMA, NGS is viewed favorably by the majority of participants, irrespective of the availability of treatments and irrespective of the screen's ability to accurately determine the type of SMA affecting the infant. This finding is in contrast to policy reviews and criteria where the absence of accurate typing and treatment for SMA were seen as fatal flaws to screening implementation (Cartwright, 2012). It is also in contrast to attitudes toward other forms of screening for SMA (PCGS and PNGS), where inability to determine type was controversial, particularly among adults with type II SMA (Boardman et al., 2017). Unlike PCGS and PNGS, which potentially involve the prevention, or termination, of lives affected by SMA (Boardman et al., 2017), NGS, through its focus on early detection, is the least emotive, and consequently the least divisive, form of screening for SMA. It has, furthermore, been identified by the SMA research community as the form of screening most likely to yield the most progress in terms of treatment development, through its concomitant increase in infants participating in clinical trials (Phan et al., 2015).
